# Infection after knee replacement: a qualitative study of impact of periprosthetic knee infection

**DOI:** 10.1186/s12891-018-2264-7

**Published:** 2018-10-02

**Authors:** Charlotte M Mallon, Rachael Gooberman-Hill, Andrew J Moore

**Affiliations:** 1 Bristol Medical School, University of Bristol, Bristol, UK; 2 National Institute for Health Research Bristol Biomedical Research Centre, University of Bristol, Bristol, UK

**Keywords:** Periprosthetic infection, Revision, Surgical treatment, Impact, Qualitative, Biographical disruption

## Abstract

**Background:**

Approximately 340,000 knee replacements are performed each year in the USA and UK. Around 1% of patients who have had knee replacement develop deep infection around the prosthesis: periprosthetic knee infection. Treatment often requires a combination of one or more major operations and antibiotic therapy. This study aimed to understand and characterise patients’ experiences of periprosthetic knee infection.

**Methods:**

Qualitative semi-structured interviews were conducted with 16 patients (9 men, 7 women; 59–80 years, mean age 72) who experienced periprosthetic knee infection and subsequent revision treatment in six National Health Service orthopaedic departments. Interviews were audio-recorded, transcribed, anonymised and analysed thematically. The concept of biographical disruption was used to frame our analysis, and four transcripts double-coded for rigour. Patients were interviewed between two and 10 months after surgical revision.

**Results:**

Participant experiences can be characterised according to three aspects of biographical disruption which we have used to frame our analysis: onset and the problem of recognition; emerging disability and the problem of uncertainty, and chronic illness and the mobilisation of resources. Participants’ experiences of infection and treatment varied, but everyone who took part reported that infection and revision treatment had devastating effects on them. Participants described use of social and healthcare support and a need for more support. Some participants thought that the symptoms that they had first presented with had not been taken seriously enough.

**Conclusions:**

Periprosthetic knee infection and its treatment can be life-changing for patients, and there is a need for greater support throughout treatment and lengthy recovery. Future work could look at preparedness for adverse outcomes, help-seeking in impactful situations, and information for healthcare professionals about early signs and care for periprosthetic infection.

## Background

In the USA and UK, approximately 340,000 primary knee replacements are performed each year [1, 2]. In 2015, 98,591 primary knee replacement procedures were performed in England and Wales alone [2]. Primary knee replacement is most commonly performed for osteoarthritis [3]. Although knee replacement often improves function and decreases pain, complications after knee replacement can include long-term pain [4], periprosthetic fracture [5] and prosthetic joint infection [6]. For some complications, revision surgery is required. Periprosthetic infection occurs when the tissues surrounding the prosthesis become infected, and it accounts for 25.2% of revision procedures after knee replacement [7] with reported incidence in the UK and USA ranging from 0.5 to 2% [6, 8, 9]. In 2015 in the UK, 6104 knee revision procedures were performed, of which nearly one quarter (1420 (23%)) were for infection [2].

Periprosthetic infection is commonly treated with either one-stage or two-stage revision. One-stage revision treatment consists of one major surgery, during which the prosthesis and infected tissues are removed before a new prosthesis is re-implanted [10]. Two-stage revision treatment consist of two separate operations where the reinsertion of a new implant is delayed allowing for additional antibiotic therapy between surgeries, sometimes via an antibiotic-impregnated cement spacer [10]. Two-stage surgical revision is the most common treatment for infection after joint replacement [11], however infection is costly to health services [12] – a revision for infection is more than three times that of an aseptic revision [13] – and two-stage revisions are more expensive than one-stage revisions with the patient undergoing two major surgical procedures [14, 15].

Previous research on infection after joint replacement highlights how infection and treatment can have profoundly negative impact on all aspects of patients’ lives [16–18]. Due to the heavy physical and psychological burden of treatment that periprosthetic infection imposes on patients and their families, there is a need for increased psychological and rehabilitative support during treatment and long-term recovery [18].

So far, research has not drawn on social science theories to extend and deepen understanding of the impact of periprosthetic infection on patients. In understanding the impact of major health and treatment events, insights from the social science of health and illness are useful, particularly regarding disruption to people’s lives, and how people negotiate and manage major change brought about by illness. Bury’s work on ‘biographical disruption’ is widely recognised as making a major contribution to the greater understanding of experiences of health and illness events. It has been used in health research to highlight how illness disrupts people’s expected life course in multiple ways, changing the structures of daily life, challenging and redefining people’s sense of identity and constructed biography [19–22]. Bury’s work has been applied in diverse health contexts, including rheumatoid arthritis [19], long-term knee pain [23], osteoarthritis [24], multiple sclerosis, cerebral palsy, blindness [25]; Meniere’s disease [26] and cancer [27, 28]. Bury describes three aspects of disruption that take place in the experience of chronic illness: onset and the problem of recognition; emerging disability and the problem of uncertainty, and chronic illness and the mobilisation of resources [19]. In exploring the biographical impact of illness, Bury [29] conceptualises the symptoms of chronic illness as having two distinct meanings: *meaning as consequence*: the problems experienced by people as a result of activity restriction, social disadvantage and their impact on daily life; and *meaning as significance*: the connotations that illnesses carry, in a cultural context. Critique of Bury’s work often focusses on these two distinct meanings and the degrees to which each is true, with the concept of ‘normal illness’ [20] set against that which is disruptive. The concept of biographical disruption is “predicated in large part on an adult-centred model of illness denoting the shift from a normal state of health to one of illness” [25]. Based on our previous work [18], periprosthetic infection may be an example of this shifting circumstance.

In this qualitative interview study, we explore the experience of periprosthetic knee infection and its impact on patients’ lives and draw on the concept of biographical disruption to sensitise us to the meanings of significance and consequences of this major health event for individuals in the context of their social worlds.

## Methods

### Study design

The study was a qualitative, interview study comprising in-depth interviews and thematic analysis.

### Eligibility and recruitment

Eligible participants were people aged 18 years and over, with periprosthetic knee infection and experience of one-stage or two-stage revision surgery. They were all patients who had received treatment at one of six participating UK National Health Service (NHS) orthopaedic departments in the 12 months before recruitment.

Between January 2016 and September 2016, 33 patients were invited to participate. A research nurse at each centre reviewed outpatient clinic lists. All eligible patients were provided with information packs and asked to complete and return a reply form to the research team indicating if they were interested in discussing participation. The research team then contacted those patients who expressed interest and arranged a mutually convenient visit to discuss the study and to conduct an interview if they agreed to participate. At that visit, patients had the opportunity to ask any further questions and were asked to provide their written, informed consent, including consent to audio-recording the interview and to publication of anonymised quotations. Once consent was provided, interviews took place at the same visit.

### Sample size

Final sample size was intended to depend on the achievement of saturation, evidenced by no new themes arising from the data [30, 31]. Saturation was achieved once 16 participants had been interviewed, and at this point data collection ceased.

### Interview process

Interviews were conducted in participants’ homes, by one of the research team’s two experienced qualitative researchers (CM and AM). The interviewers were not previously known to the participants. Topic guides were developed in collaboration with the research unit’s patient and public involvement forum [32]. Topic guides included key questions, with probes and prompts used where appropriate to allow for flexibility and to ensure that participants had the opportunity to discuss subjects they deemed important. Questions covered experience of periprosthetic infection, revision surgery and post-operative care, impact of infection and subsequent treatment, and concerns and expectations for the future. Interviews lasted from 33 to 95 min (mean 67 min).

### Data analysis

A team science approach was used to ensure robust analysis, whereby our research was collaboratively conducted by a small team of researchers [33]. Interviews were transcribed, anonymised, with all identifying information removed or replaced with pseudonyms, and imported into the qualitative data management software QSR NVivo [34]. Using a thematic approach^,^ the researcher (CM) read and re-read the transcripts, inductively and deductively coded them and sorted coded data into themes. Deductive coding involved working “down” from pre-existing understandings from previous research, which sensitised us to the data [35]. A second researcher (AM) double-coded four of the 16 transcripts, and the study team met to discuss and agree codes and themes [36]. Bury describes three aspects of disruption which we have used to frame our analysis: onset and the problem of recognition; emerging disability and the problem of uncertainty, and chronic illness and the mobilisation of resources [19]. Within this framework, we also consider both the meaning as consequence and the meaning as significance, for the symptoms and treatment of periprosthetic infection [29].

Ethical approval was granted by NRES Committee South West - Exeter (14/SW/0072).

## Results

The sample consisted of 9 patients who received one-stage revision treatment, and 7 patients who received two-stage revision treatment; 9 men and 7 women, aged 59–80 years (mean age 72 years) (Table 1).

**Table 1:** Sample characteristics

Pseudonym	Sex	Age range	Revision Procedure
Winston	Male	71–80	Two-stage
Delia	Female	61–70	Two-stage
Harry	Male	71–80	One-stage
Shirley	Female	71–80	Two-stage
Hilary	Female	71–80	Two-stage
Margaret	Female	71–80	Two-stage
Brian	Male	71–80	Two-stage
Louisa	Female	61–70	One-stage
Peter	Male	61–70	Two-stage
Terry	Male	71–80	One-stage
Derek	Male	71–80	One-stage
Hazel	Female	51–60	One-stage
Pam	Female	71–80	One-stage
Doug	Male	61–70	One-stage
Lloyd	Male	61–70	One-stage
Jimmy	Male	61–70	One-stage

For 14 patients, this was their first revision surgery. One had received a previous two-stage revision. With the exception of one participant whose primary knee replacement was the result of a fracture, participants’ primary replacements had been elective procedures to relieve pain associated with osteoarthritis.

The results highlighted participants’ varied experiences, in particular regarding infection onset and subsequent treatment. Infection onset occurred over periods ranging from immediately to 19 years after their primary surgery. All participants felt that infection was life-changing, describing it as “devastating”, “traumatic” and causing a “considerably restricted life”. Patients’ narratives suggest that the experience of infection can be understood as a temporal situation, which can be mapped onto Bury’s three aspects of disruption: Onset and the problem of recognition; emerging disability and the problem of uncertainty; and chronic illness and the mobilisation of resources. Participants discussed the ways in which infection and its treatment impacted on, and disrupted the life course within each aspect.

Our analysis suggests that the experience of infection and its treatment causes patients to negotiate major change brought about by this illness event, and to redefine their biographies. Participants described the disruption of taken-for-granted daily routines, uncertainty about the impact and course of infection and treatment, and anxiety and fear about the future. Illustrative quotations are referred to throughout the text and are presented in Tables 2, 3 and 4.

**Table 2:** “Onset and the problem of recognition” quotations and themes

Recognition of infection	“I was annoyed, I was annoyed more than anything that I told so many people that I didn’t think it was right and I trusted that they knew better than me because I’m not a doctor or a surgeon” Hazel (1)
	“Every time the answer was ‘it can take up to two years to get better’…so all this wonderful Nirvana I were expecting never came about” Hazel (1)
	“Well just want to get right, get right, but um, it was gradually getting worse and worse and worse and at a different stage I thought ‘I’m not gonna get out of here’ I thought ‘I’ve come to my end’ cos that’s how I felt” Delia (2)
	He said, “‘I’m not giving antibiotics. We give too much of that out.’ So anyway it got worse…and then the knee became very painful. I went back over the surgery and, I asked to see a different doctor, who took one look at it and said, ‘It’s very badly infected.’” Jimmy (1)
	The professionals in there appeared to not take the infection seriously enough, and the GPs also – which are normally your first point of call, didn’t take it seriously. Jimmy (1)
Infection onset	“I can’t believe it just because I had no, during the day no inclination that anything was wrong with my knee at all… Really, really strange but I mean I don’t know if that’s how infections happen I don’t know, or if you have a build up to an illness, I never had a cold, I wasn’t ill.” Delia (2)
	“…it was very painful. In fact, it was so painful, I couldn’t even walk for many months and it was decided I got an electric scooter.” Harry (1)
	“I didn’t really know what was happening, because I just thought it was the poison coming out and I’d be better, you see, so I was wrapping it up and wrapping it up, and in the end I thought, ‘Well, you know, it’s not stopping. I better go and speak to the doctor…’” Derek (1)
	“I was in horrendous pain and my knee was literally twice the size of my other one. [hmm] And I knew that couldn’t be right. And no amount of icepacks was making any difference. [no] And the painkillers. I was on about five different painkillers and I mean I’ve got a high pain threshold but I, I just, I just couldn’t get the pain, you know, I was climbing the walls, really.” Louisa (1)
	I got out the bed one morning…went to walk to the bathroom and my knee just went, just let me down. Brian (2)
Preparing for diagnosis	“Well, I’ve always been very active and worked all my life, you can say, and I’m - I get up and go and I like to do things. I never thought I’d be wrong. I thought, ‘I’ll do the physio and I’ll do ...’ you know. It didn’t’ occur to me that it would go wrong.” Pam (1)
	“They did (mention the possibility of infection). Yes, they did say that, but, they sort of, did it so gently and so lightly, ‘There’s always that risk, but, you know, things will be expected to run normally. We’re not expecting any problems,’ so this came as a bit of a shock to me, actually.” Margaret (2)
	“Well, yeah, it was. I mean, I was really annoyed with [surgeon], because, when I went up to theatre to have it manipulated I was on the trolley, coming out of the lift into the theatre area, and he came to me and said, ‘If you’d put more effort in with the physios, there’d be no need for this.’” Peter (2)
	I just thought, ‘This is nearly two years out of my life and at my age [yeah] it, it’s not on.’ After everything I’d been through as well previously with, you know, different operations [hmm]. I was fuming. I thought, ‘If I see the guy I shall hit him.’ Louisa (1)
	“Well, you see, it seems like they’re in denial because they have this knowledge, they know how serious the infections are … some terrible stories.… But it seems like they let it get to such a bad state first of all before they do any of that. Whereas after, erm, joint surgery, if that’s how serious an infection can be, it ought to be acted on earlier on, really.” Jimmy (1)
	“I paid privately to have a private consultation to see him … I needed to have some sort of answers fairly soon for my own peace of mind and, er, he arranged for aspiration and it came back fairly quickly, ‘You’ve got a, an infection’. So at least then to some extent I was quite happy because I knew what the problem was.”

**Table 3:** “Emerging disability and the problem of uncertainty” quotations and themes

Burden	“I got down a lot as well because I had to have so long off work it was six months. So I was on statutory sick pay and having to claim rent rebate and stuff, it were a nightmare to me … it’s statutory sick pay 29 pound a week so I mean what’s that when your rent’s 96? So the finances things got me. I had to go into my overdraft and that’s something I just don’t, you know that’s your rainy day money. Because living on your own you do live hand to mouth, you don’t have savings, but I can still stand on my own two feet, pay all my bills and buy all my stuff what I need, so that’s why I work every Sunday, to make sure I can do that but of course, for six months there were none of that.” Hazel (1)
	“Well you have to rely on other people don’t you? [Yeah] When you’re stuck with a brace on your leg, it’s like having a broken leg in a, in a cast for a year isn’t it? [Yep] Yeah, you are dependent on everyone really.” Delia (2)
	“As you can imagine, lots of things you can’t do, for one thing I couldn’t drive a car for a year” Delia (2)
Antibiotic therapy	“Actually I wasn’t too bad, I mean some people have lots of side effects but I was, I was alright.” Delia (2)
	“I think I lost about a stone and a half in weight [laughter] [mmm]. I really felt ill [yeah, yeah]. Erm, that was the thing I didn’t like about it and erm... but it did the trick” Shirley (2)
	“When this doctor came in and said you should be able to go home tomorrow he came back to tell me I couldn’t because I had to stay on these antibiotics again. Now this was three weeks and I was actually crying and saying I really can’t deal with this diarrhoea and stuff and nobody would tell me ‘why have I got to keep having them?’.” Hazel (1)
Trust	“When he [surgeon], when he said, ‘I think I might have to go in and have another look,’ I knew what he meant. It’s going to be a revision. [hmm] And I thought, ‘He’s, he’s not going to do this. If it’s - if I’ve got to have it done again I’m not letting him touch me.’” Louisa (1)
	“Trust them? [Yeah] Because we think they know, don’t we? We think they’re wise.” Pam (1)
	“I have been through it over the last seven years, believe you, me but this last knee, so far, has been brilliant. [Surgeon] knows what he’s doing.” Harry (1)
	“I really felt that he was doing his best for me, I really did, you know, I thought so much of him, I had so much confidence in him, the way he dealt with everything … it was quite incredible actually, he was so good on that, erm, that, er, that I didn’t query anything. I was just in his hands, I put myself in his hands.” Derek (1)
	“I found an absolutely brilliant surgeon and I wouldn’t go anywhere else, wouldn’t go anywhere else, if he won’t do I’ll stick without [yeah] because I trust him.” Winston (1)
Two-stage revision	“Well film someone who’s – who’s had that surgery in – in its – in its different stages, when you’ve just had it in, the first time you get up and use it and – and show you how his body is a bit wobbly and you know, and all this type of thing. Because if people can see for themselves that it’s possible and it’s good, because you don’t know it is, because there’s nothing, I was given no information on how I should react to it” Winston (2)
	“I spent … March, April, May, June, [mmm] not being able to do anything” Shirley (2)
	“Well, I thought it was gonna be a nightmare but, I mean, I was on crutches [yeah]. I’m still on crutches, on one crutch, and, er, I’m still wearing a, a brace on my leg, when I’m outside walking round. Erm, but, obviously, the wife’s had to drive all the, all the time [yeah]. Erm, I haven’t been able to do things I want to do. Erm, I’m retired, but I did intend to carry on doing a few jobs for, you know, people that I know well [yeah]. Erm, so, basically, you know, it has stopped me doing a lot of the things that … I mean, I’d just retired two months before the operation, so I’ve not really enjoyed retirement, because I’ve been restricted in what I can do.” Peter (2)
	“Oh the whole thing’s quite frightening but I mean I can be negative but my husband’s so negative I’m determined to not to be negative you know what I mean, I think I can’t put up with all of this.” Hilary (2)

**Table 4:** “Response to infection and treatment, and the mobilisation of resources” quotations and themes

Social support	“But, as I say, it’s, err – and of course it means you don’t go off to see your parents and your family and your children half as much as you normally would. They come to you, which is wonderful, but, I mean, it’s putting them at difficulties sometimes, when on many occasion we go to visit them, you know.” Margaret (2)
	“What was going to happen after I came out of hospital? Hubby’s useless, absolutely useless, I’m there for him he isn’t there for anybody else.” Hilary (2)
Changing the physical environment	“I were frightened when I were left on my own and there were nobody in, I don’t know what I was frightened of but I was frightened of irrational things. What if house catches fire downstairs?” Hazel (1)
	“I have trouble with my bath but that’s not their fault. It’s a, a shower bath and I can’t have a seat on or anything because it’s too wide. But it’s a big corner and I sit on there and I swing my legs.” Pam (1)
	“I try and go up to my daughter, she’s got a walk-in shower” Brian (2)
Clinical support	“It’s just that to start with I think I was feeling so low and so very unwell, I really felt neglected.” Margaret (2)
	“One would tell you one thing, one would tell you another and I think again this all contributed to my feeling quite low and when I got home I came home thinking right I will get myself better now, I’m home now.” Hazel (1)
	“No, I’ve not had any physio, no. It was, erm, before I came out it was a matter of, let me think, before I came out.” Doug (1)
	“Yeah, they [physiotherapists] were around every day while I was in the hospital.” Brian (2)
	“They [physiotherapists] didn’t come to me. In fact, they never came to me. All they brought was that ice bucket thing ... and I didn’t actually know how to do it.” Pam (1)
	“Six weeks it was before I could see a physio. Well, luckily, they gave me some exercise sheets at the hospital and luckily, I’m the sort that would do it.” Shirley (2)
Life after periprosthetic infection	“I had discomfort, I couldn’t walk very well so I went to see [surgeon] and err, he said I think you ought to have a knee replacement so that’s what happened” Terry (1)
	“But I, I just thought, ‘This is nearly two years out of my life and at my age it’s not on” Louisa (1)
	“I’m 80 in September, and I’m not young, and I can’t expect to be playing football and cricket and running around, and the only thing I wish I could get on the floor and play with the grandchildren and their games sometimes, but that’s not the point, the point is that I’ve accepted my age, and I don’t look for people running around after me” Derek (1)
	“What’s the next stage, what’s going to happen … am I going to get infection back, you know, there’s only so much your knee can take” Delia (2)

### Onset and the problem of recognition (Table 2)

The onset of infection marked a biographical shift from an expected normal course of recovery after knee replacement, to one which was abnormal. Participants described the onset of infection in terms of length of time after surgery, diverse sensations and impact. The onset of infection did not appear to follow a predictable path, and varied from “immediately” after primary replacement (participants felt it never got better) to 19 years afterwards. Although some participants recalled discussing the risk of infection before they had their primary replacement, most did not recollect such conversations. Those participants who could recollect a discussion reported being informed that along with many other factors, infection was a “risk” of joint replacement, however despite this, they still felt largely unprepared for infection.

One participant reported having no indication that anything was wrong until she was suddenly unable to place her foot on the floor. Two patients felt so ill after their infection had manifested, they thought that they would die: one of these participants reported sudden onset at 19 years afterwards, the other described how she never reached her expected “Nirvana” of two years to fully recover after the primary replacement, and despite questioning clinicians, was eventually admitted to hospital by emergency ambulance after collapsing at home. Some participants described more obvious signs that something was wrong, such as red, warm and leaking wounds or scars; others described swelling or the presence of sinus tracts or lumps. Although some described severe pain, others did not.

The process of recognising and reporting the illness to a healthcare professional was problematic for all participants, and routes to diagnosis varied. The length of time between first reporting that there was a problem, and treatment at one of the six orthopaedic departments, ranged from immediately to five years. Some healthcare professionals recognised that participants’ symptoms might indicate infection, and made a diagnosis quickly. For some participants diagnosis was prolonged as their symptoms were not immediately thought to be indicative of infection. Participants who experienced a slower route to diagnosis were dissatisfied and felt that their concerns had not been taken seriously enough by healthcare professionals. Although some patients were insistent with healthcare professionals that there was a problem, others felt frustrated with themselves for not “speaking up” when they had intuitively felt that something was wrong. Patients’ reasoning for not challenging healthcare professionals about their symptoms included viewing surgeons as expert and therefore trusting, or having “faith” in their abilities, not wanting to be perceived as “bolshie or rude or pedantic”, not knowing appropriate questions to ask, and fearing they would “hold everything up” by challenging decisions. Consequently, some participants felt that their pursuit of referral had been considerably delayed, and that if their infection had been diagnosed sooner, the impact on their life may have been less severe.

Participants described conflicting reactions to their diagnoses. The diagnosis of infection was a shock to some participants. Despite a late diagnosis some participants reported feeling “pleased” and “relief” that treatment was imminent. Others felt “disappointed”, “unlucky” or “annoyed”. One patient described feeling vindicated, and described how he had felt the surgeon who conducted his primary knee replacement “blamed” him for a lack of improvement after surgery. At the point of diagnosis, some patients felt uncertain about how the infection might progress, and what the subsequent treatment might entail. Despite a firm diagnosis the cause of the infection was often unknown, and participants reported feeling shocked, and a mixture of fear and relief at the point of diagnosis, but also concerned and uncertain about their future.

For the participants in this study, the onset of infection was not perceived as a “normal” part of their biography. It was a shock, and a source of uncertainty and anxiety, about its cause, the treatment and the prognosis. The onset and diagnosis of infection was traumatic for many participants, and something for which they were unprepared.

### Emerging disability and the problem of uncertainty (Table 3)

Surgical and antibiotic treatment for infection profoundly disrupted participants’ everyday lives in multiple ways, reinforcing a biographical shift from a perceived normal to an altered situation and sense of self. Participants’ responses to antibiotic therapy varied. Not all patients felt unwell during antibiotic treatment and most did not experience any adverse effects. However, some patients experienced unpleasant and distressing side effects, including nausea, sickness, loss of appetite, diarrhoea and weight loss. Describing her low mood and distress after three weeks of unpleasant side-effects, one patient felt that she could no longer cope with her treatment regimen.

Surgical treatment for infection impacted on patients’ physical mobility and function, and some participants experienced associated social, psychological and financial burdens. Participants lived with mobility and lifestyle limitations including being unable to drive, work, sleep, carry out domestic duties, or walk without pain. Leisure activities, social engagements and visits to family were often cancelled, and participants were unable to continue their previous lifestyle as a result of severe immobility. Infection also had a financial impact on participants. One participant cancelled her holiday, losing money on flights and accommodation; another felt “lucky” that he had completed all of his mortgage payments in the same month as his operation, believing that he would have been forced to sell his house otherwise as he could no longer work. This profound physical, social, psychological and financial disruption led to participants describing their lives as “on hold”.

Participants spoke of the trust and faith they placed in their surgeon. Some patients retained a trust in their surgeon especially if the same surgeon that performed their primary knee replacement also performed the revision operation. However, others lost trust in the surgeon who conducted their primary replacement. One participant described how she did not “trust” her original surgeon to perform a revision operation, because she felt that her insistence that something was wrong had not been taken seriously enough. Additionally, she described an initial conversation with her surgeon in which she was assured she would be able to walk, and engage in sporting activity after knee replacement: activities she was unable to do. In contrast, one participant insisted the same surgeon who had performed his primary replacement also performed his subsequent three revisions.

Participants expressed a sense of uncertainty about the eradication or the return of infection. Many patients reported that a recurrence of infection was their main concern for the future: some patients sought post-operative assurances from their surgeons about the “percentage” or “likelihood” of eradicating infection completely; other patients described their hopes that the infection would not return. This sense of uncertainty led to patients feeling “terrified” and “very anxious” of the impact that a recurrent infection and further surgery would have on their lives.

The significance of participants’ treatment was neither downplayed, nor thought to be a “normal” part of their biography. For the participants in this study, the treatment of infection was a complex, and often lengthy process. In line with this, the consequences of participants’ treatment for infection were severe for all participants, with profound physical, social, psychological and financial disruption, which profoundly changed their daily lives. Participants described withdrawal from their valued activities and relationships as a result of treatment, which prevented them from doing things they had previously enjoyed.

### Response to infection and treatment, and the mobilisation of resources (Table 4)

Participants’ responses to the disruption of infection and treatment involved a restructuring of their personal and social involvements. Participants experienced a sense of hopelessness, and felt that their personal identity had changed over the course of the infection and treatment: from a dependent, capable self to an uncertain, increasingly dependent self for whom the infection “takes over”. Participants found that taken-for-granted activities they were once easily able to undertake were no longer possible. Most participants felt that their relationships with family and friends had been disrupted, either because of mobility restrictions, tiredness, or embarrassment in social situations. One participant described how he felt he placed an unfair burden on his wife who missed out on social and leisure opportunities. Another participant described how she felt she was a burden to her older children and had tried to avoid socialising with them.

In terms of mobilising resources to face a new and unexpected situation, participants relied mostly on support from social networks including family, friends and neighbours in relation to cooking meals, shopping for food, undertaking domestic duties, and accessing their own home. The presence of a supportive social network was fundamental to all participants’ recovery, and participants described how maintaining their social network was of great concern.

Participants described how they made adaptations to their physical home environment, in order to manage their recovery after revision treatment. Some moved out of their home to live with others during their recovery. One patient moved in with her daughter, during her post-operative recovery, but became anxious when left alone in the house due to her immobility. As self-care became difficult, participants’ spouses or children helped them to maintain their personal hygiene. Participants also discussed being unable to shower, or creating complex routines to enable them to do so, including travelling to a family member’s home to use a walk-in shower.

In terms of medical support and resources, some participants reported needing more care and support with their post-operative and long-term recovery from Primary Care doctors and allied health professionals, in the period after surgical revision treatment. One participant felt that the lack of psychological support after her discharge from hospital had led to her low moods. Another patient described an absence of support from both clinicians and her spouse led to her being unable to manage her medication once discharged. Post-operative physiotherapy varied between treatment centres. Some participants reported having minimal physiotherapy input, or none at all, whilst others reported satisfactory post-operative physiotherapy. However, patients’ experiences varied. Only one patient received a course of hydrotherapy. Another described having only one physiotherapy appointment in her eight-day recovery in hospital, at which she was shown how to use cold therapy ice pads, but still felt uncertain of how to use them.

There was a stark contrast between participants’ expectations of primary knee replacement, and the reality of life after periprosthetic infection. Many participants described their altered life course in ways that inferred an acceptance of their situation as a new normal, rather than an illness event. Participants discussed feelings of uncertainty, about the recurrence of infection, or requiring lifelong antibiotic medication. When asked about their original preconceptions of primary knee replacement, participants largely spoke of reduced pain, walking without discomfort, and a return to being “fit and active”. In contrast to their preconceptions, one patient had experienced a “superb” recovery after his primary replacement until his infection four years later, however at the time of interview was unable to walk without discomfort, and described a painful and stiff joint: he was fearful that his infection had not cleared. One patient had experienced a “terrible” and lengthy recovery after her primary replacement, and despite feeling satisfied with the revision operation, felt that she had lost two years of her life.

The frustration of coping with either pain or immobility in their daily lives contributed to patients’ low mood. The impact of both infection and treatment on wider family and significant relationships was profound, with some participants acknowledging a fear of dependency. Participants’ responses to the infection and treatment also expressed a sense of fragility, using phrases such as “I’m hopeless now”, “I do need answers now because I can’t carry on”, and “It makes me feel useless.”

Participants’ narratives suggest that both the meanings as significance and consequence of symptoms and treatment for periprosthetic infection create a biographically abnormal and profoundly disruptive experience for patients, wherein social identities are challenged and redefined.

## Discussion

People diagnosed with periprosthetic infection faced an altered and unexpected situation, in which they experienced profound disruption to their life course according to Bury’s theory [19]. Infection and treatment appeared to derail people’s sense of a planned or anticipated biographical trajectory, which they found distressing. Their distress was particularly related to difficulties in getting a diagnosis of infection; a relatively sudden lack of mobility from onset of infection and throughout treatment; an uncertainty about their future; and a forced withdrawal from their social worlds. Bury’s theory provides a useful framework through which to view the narratives of patients, through each of the three aspects of disruption that take place in patients’ experience of chronic illness.

Participants made sense of their experiences of infection through the narratives that they shared, giving meaning to the events of infection that disrupted the structures of their everyday lives, and the foundations that underpin these structures [19]. Narratives often began with an uncertain, difficult or traumatic diagnosis during which some patients felt that signs of infection were “not taken seriously enough” and should have been acted upon earlier. This was followed by a disruption of taken-for-granted behaviours, and persistent uncertainty during their treatment and rehabilitation, for which they were largely unprepared. Not all participants could recall discussing the risks of infection with their doctor, or receiving information about infection, before their primary joint replacement. While this apparent lack of information may have enabled patients to remain calm and positive about their primary joint replacement, it may also have increased the likelihood of patients’ lack of preparedness when infection was later diagnosed. However, it is also possible that patients did not expect to be one of the 1% of primary knee replacement patients who develop an infection.

Periprosthetic infection can be life-changing, both physically and psychologically, and placing pressure on patients’ social and supportive relationships. The impact on participants’ mobility and physical function restricted their ability to participate in social roles and events as they had previously, subsequently affecting their sense of personal identity. Some participants were forced to adapt their home environments or move home entirely to cope with their recovery. Participants described disruption to their personal identity and relationships with others, in a context where infection and treatment precluded their former positive experiences and meanings [19, 22]. Withdrawal from social relationships as well as increasing social isolation are important features of chronic illness [37]. Increasing social isolation and dependency on their families and wider social networks, led participants to describe how they felt they were being a burden to those who cared for them. Throughout participants’ narratives uncertainty is evident - at diagnosis, treatment, rehabilitation and in concerns about eradication or return of infection. In their narratives, participants incorporated this difficult and traumatic illness event into their biography, but they often remained uncertain regarding their future. The patients in this study described a persistent need for support throughout diagnosis, treatment and recovery. Despite the relatively small number of cases of periprosthetic infection each year, the impact on patients’ lives is disproportionately adverse.

Despite its widespread application, biographical disruption has been debated [24, 25]. Yet notwithstanding these alternate views, it remains a powerful sensitising concept to the meanings of significance and consequences of periprosthetic infection for individuals in the context of their social worlds. In understanding the significance and consequence of periprosthetic infection, we suggest that its onset and treatment represents a completely unexpected and anomalous event which profoundly disrupts the lifecourse. This is the first study to explore the impact of one-stage and two-stage revision treatment for periprosthetic knee infection. Previous qualitative work has explored periprosthetic hip infection [18] and surgical site infection [38]. Our study also indicates that infection impacts on all aspects of patients’ lives [16–18], but also explored the relationship between the experience of infection and treatment, and biographical disruption. Bury’s work on biographical disruption focuses on the onset and the perception of illness [19]: our work extends this by also focusing on the treatment of, and recovery from illness, which in the context of prosthetic joint infection arguably has the greatest impact on patients. While we acknowledge that the onset of periprosthetic infection is distressing and impactful for patients, they remain largely unprepared for the potentially lengthy and complex treatment process and recovery period. This impacts on patients’ ability to mobilise resources in order to cope with their treatment and recovery, and the participants in this study described feeling irrational, anxious and in low mood, and very aware of the burden they placed on their supportive networks. Our findings draw parallels with work describing how feelings of hopelessness are reported among patients with surgical site infections [38]. Both the loss of identity and independence established in this study are similar to those found in other chronic illnesses [39, 40]. The unpredictability and uncertainty of diagnosis and treatment of periprosthetic infection draw parallels with patients’ perceptions of multiple sclerosis [41], HIV [42–44], and cancer [45]. The disruption to the life course that patients in this study experienced is similar to those experiencing HIV and cancer diagnoses [46–48]. Uncertainty is widely recognised in qualitative studies of illness experience literature, and patients’ uncertainty is greatest during the diagnosis phase and when outcomes are unknown or unpredictable [49].

This study provides new information about patients’ experiences of periprosthetic knee infection. Saturation was achieved in the sample of 16 patients from six UK NHS orthopaedic centres, and we took care to ensure rigour in analysis through a double coding and team science approach. We acknowledge that it is possible that the inclusion of more patients from additional study centres may have elicited supplementary findings. We also acknowledge that a study limitation, common to all research which employs opt-in consent is that there is an inherent self-selection bias, however, achievement of saturation gives us confidence that the sampling was appropriate in quantity and breadth. The study was conducted in the UK context, but we suggest that the experience of infection after knee replacement resonates with other contexts, including social impact and need for care. Interviewing participants between two and 10 months after revision surgery may have introduced recall bias but this approach gave the study the opportunity to explore the longer-term impact of infection, treatment and recovery.

## Conclusions

The impact of periprosthetic infection is wide-ranging, and research to date has not paid sufficient attention to the experiences of people who have this complication after knee replacement. Participants within this study described a disruption to their everyday behaviours, a loss of identity, growing dependency on families and social support networks, as well as uncertainty about their futures. As such we suggest that periprosthetic knee infection may be described as an assault on patients’ physical self, sense of self and life course. Further research into the impact of infection might take a longitudinal, prospective approach to explore recovery and change over time in more detail. Our findings lead us to suggest that clinicians in primary, secondary and community care should be supported to provide consistency in care, not only through conveying the importance and urgency of infection diagnosis, but also by being vigilant to the early warning signs and symptoms of periprosthetic infection. Indeed, early diagnosis of periprosthetic infection maximises the chance of prosthesis retention [50]. Future research could focus on patient preparedness for adverse outcomes after joint replacement, help-seeking in the event of periprosthetic infection, and clinician support in the early recognition of periprosthetic infection.

## References

[1] Cram P, Lu X, Kates SL, Singh JA, Li Y, Wolf BR (2012). Total knee arthroplasty volume, utilization, and outcomes among Medicare beneficiaries, 1991-2010. JAMA.

[2] National Joint Registry Reports. National Joint Registry for England, Wales and Northern Ireland, 13^th^ annual report. 2016. http://www.njrcentre.org.uk/njrcentre/Reports,PublicationsandMinutes/Annualreports/tabid/86/Default.aspx. Accessed 25 Jan 2016.

[3] Dieppe P, Basler HD, Chard J, Croft P, Dixon J, Hurley M (1999). Knee replacement surgery for osteoarthritis: effectiveness, practice variations, indications and possible determinants of utilization. Rheumatology.

[4] Beswick Andrew David, Wylde Vikki, Gooberman-Hill Rachael, Blom Ashley, Dieppe Paul (2012). What proportion of patients report long-term pain after total hip or knee replacement for osteoarthritis? A systematic review of prospective studies in unselected patients. BMJ Open.

[5] Whitehouse MR, Mehendale S (2014). Periprosthetic fractures around the knee: current concepts and advances in management. Curr Rev Musculoskelet Med.

[6] Blom AW, Brown J, Taylor AH, Pattison G, Whitehouse S, Bannister GC (2004). Infection after total knee arthroplasty. Bone & Joint J.

[7] Bozic KJ, Kurtz SM, Lau E (2010). The epidemiology of revision total knee arthroplasty in the United States. Clin Orthop Relat Res.

[8] Kurtz SM, Ong KL, Lau E, Bozic KJ, Berry D, Parvizi J (2010). Prosthetic joint infection risk after TKA in the Medicare population. Clin Orthop Relat Res.

[9] Poss R, Thornhill TS, Ewald FC, Thomas WH, Batte NJ, Sledge CB (1983). Factors influencing the incidence and outcome of infection following total joint arthroplasty. Clin Orth Rel Res.

[10] Nazarian David G, Jesus Dino de, McGuigan Francis, Booth Robert E (2003). A two-stage approach to primary knee arthroplasty in the infected arthritic knee. The Journal of Arthroplasty.

[11] Kapadia BH (2016). Periprosthetic joint infection. Lancet.

[12] Kapadia BH, McElroy MJ, Issa K, Johnson AJ, Bozic KJ, Mont MA (2014). The economic impact of periprosthetic infections following total knee arthroplasty at a specialized tertiary-care center. J Arthroplast.

[13] Kallala R. F., Vanhegan I. S., Ibrahim M. S., Sarmah S., Haddad F. S. (2015). Financial analysis of revision knee surgery based on NHS tariffs and hospital costs. The Bone & Joint Journal.

[14] Parkinson RW, Kay PR, Rawal A (2011). A case for one-stage revision in infected total knee arthroplasty?. Knee.

[15] Kunutsor Setor K., Whitehouse Michael R., Lenguerrand Erik, Blom Ashley W., Beswick Andrew D. (2016). Re-Infection Outcomes Following One- And Two-Stage Surgical Revision of Infected Knee Prosthesis: A Systematic Review and Meta-Analysis. PLOS ONE.

[16] Cahill JL, Shadbolt B, Scarvell JM, Smith PN (2008). Quality of life after infection in total joint replacement. J Orthop Surg.

[17] Kunutsor Setor K., Beswick Andrew D., Peters Tim J., Gooberman-Hill Rachael, Whitehouse Michael R., Blom Ashley W., Moore Andrew J. (2017). Health Care Needs and Support for Patients Undergoing Treatment for Prosthetic Joint Infection following Hip or Knee Arthroplasty: A Systematic Review. PLOS ONE.

[18] Moore Andrew J, Blom Ashley W, Whitehouse Michael R, Gooberman-Hill Rachael (2015). Deep prosthetic joint infection: a qualitative study of the impact on patients and their experiences of revision surgery. BMJ Open.

[19] Bury M (1982). Chronic illness as biographical disruption. Sociol Health Illn..

[20] Williams S (2000). Chronic illness as biographical disruption or biographical disruption as chronic illness? Reflections on a core concept. Sociol Health Illn.

[21] Bury M (1991). The sociology of chronic illness: a review of research and prospects. Sociol Health Illn..

[22] Charmaz K (1983). Loss of self: a fundamental form of suffering in the chronically ill. Sociol Health Illn..

[23] Morden A, Jinks C, Ong BN (2015). Temporally divergent significant meanings, biographical disruption and self-management for chronic joint pain. Health (London)..

[24] Sanders C, Donovan J, Dieppe P (2002). The significance and consequences of having painful and disabled joints in older age: co-existing accounts of normal and disrupted biographies. Sociol Health Illn..

[25] Larsson AT, Grassman EJ (2012). Bodily changes among people living with physical impairments and chronic illnesses: biographical disruption or normal illness?. Sociol Health Illn..

[26] Bell SL, Tyrrell J, Phoenix C (2016). Ménière's disease and biographical disruption: where family transitions collide. Soc Sci Med.

[27] Leveälahti H, Tishelman C, Öhlén J (2007). Framing the onset of lung cancer biographically: narratives of continuity and disruption. Psychoncology.

[28] Hannum SM, Rubinstein RL (2016). The meaningfulness of time; narratives of cancer among chronically ill older adults. J Aging Stud.

[29] Bury M, Anderson R, Bury M (1988). Meaning at risk: the experience of arthritis. Living with chronic illness. The experience of patients and their families.

[30] Glaser BG, Strauss AL (1967). The discovery of grounded theory: strategies for qualitative research.

[31] Guest G, Bunce A, Johnson L (2006). How many interviews are enough? : An experiment with data saturation and variability. Field Methods.

[32] Gooberman-Hill Rachael, Burston Amanda, Clark Emma, Johnson Emma, Nolan Sharon, Wells Victoria, Betts Lizzy (2013). Involving Patients in Research: Considering Good Practice. Musculoskeletal Care.

[33] Cooke NJ, Hilton ML, National Research Council (2015). Enhancing the effectiveness of team science.

[34] NVivo qualitative data analysis Software. QSR international Pty ltd. Version. 2012:10.

[35] Ezzy D (2002). Qualitative analysis. Practice and innovation.

[36] Barry CA, Britten N, Barber N, Bradley C, Stevenson F (1999). Using reflexivity to optimize teamwork in qualitative research. Qual Health Res.

[37] Strauss A, Glaser B (1975). Chronic illness and the quality of life.

[38] Andersson AE, Bergh I, Karlsson J, Nilsson K (2010). Patients’ experiences of acquiring a deep surgical site infection: an interview study. Am J Control.

[39] Lempp H, Scott D, Kingsley G (2006). The personal impact of rheumatoid arthritis on patients' identity: a qualitative study. Chronic Illn.

[40] Sutanto B, Singh-Grewal D, McNeil HP, O'Neill S, Craig JC, Jones J, Tong A (2013). Experiences and perspectives of adults living with systemic lupus erythematosus: thematic synthesis of qualitative studies. Arthritis Care Res.

[41] Dennison Laura, McCloy Smith Ellen, Bradbury Katherine, Galea Ian (2016). How Do People with Multiple Sclerosis Experience Prognostic Uncertainty and Prognosis Communication? A Qualitative Study. PLOS ONE.

[42] Burchardt M (2010). “Life in brackets”: biographical uncertainties of HIV-positive women in South Africa. Forum Qual Soc Res.

[43] Davies ML (1997). Shattered assumptions: time and the experience of long-term HIV positivity. Soc Sci Med.

[44] Weitz R (1989). Uncertainty and the lives of persons with AIDS. J Health Soc Behav.

[45] Nanton V, Munday D, Dale J, Mason B, Kendall M, Murray S (2016). The threatened self: considerations of time, place, and uncertainty in advanced illness. Br J Health Psychol.

[46] Ciambrone D (2001). Illness and other assaults on self: the relative impact of HIV/AIDS on women’s lives. Sociol Health Illn..

[47] Reeve J, Lloyd-Williams M, Payne S, Dowrick C (2010). Revisiting biographical disruption: exploring individual embodied illness experience in people with terminal cancer. Health (London).

[48] Navon L, Morag A (2004). Liminality as biographical disruption: unclassifiability following hormonal therapy for advanced prostate cancer. Soc Sci Med.

[49] Mishel M (1988). Uncertainty in Illness. J Nurs Schol.

[50] Schoifet SD, Morrey BF (1990). Treatment of infection after total knee arthroplasty by debridement with retention of the components. J Bone Joint Surg Am.

